# Enumerating genotypic diversity and host specificity of G*iardia* in wild rodents around a New York watershed

**DOI:** 10.1016/j.ijppaw.2024.100995

**Published:** 2024-09-19

**Authors:** Matthew H. Seabolt, Kerri A. Alderisio, Lihua Xiao, Dawn M. Roellig

**Affiliations:** a Division of Foodborne, Waterborne, and Environmental Diseases, National Center for Emerging and Zoonotic Infectious Diseases, Centers for Disease Control and Prevention, Atlanta, GA, 30329, USA; b Water Quality and Innovation, Bureau of Water Supply, New York City Department of Environmental Protection, Valhalla, NY, 10595, USA; c Center for Emerging and Zoonotic Diseases, College of Veterinary Medicine, South China Agricultural University, Guangzhou, 510642, Guangdong, China; d Guangdong Laboratory for Lingnan Modern Agriculture, Guangzhou, Guangdong, 510642, China

**Keywords:** *Giardia*, Molecular characterization, Host specificity, Rodents, Watershed monitoring

## Abstract

*Giardia* is a genus of flagellated protozoans that parasitize the gastrointestinal tract of humans and wildlife worldwide. While *G. duodenalis* is well-studied due to its potential to cause outbreaks of diarrheal illness in humans, other *Giardia* species from wildlife have been largely understudied. This study examines the occurrence, host specificity, and genotypic diversity of *Giardia* in wild rodents living within the New York City water supply watershed. A novel nested PCR assay targeting the 18S ssu-rDNA gene is introduced, which captures nearly the entire gene for improved species-level determination versus existing molecular typing methods. Molecular characterization of 55 *Giardia* specimens reveals at least seven novel lineages. Phylogenetic analysis indicates a close relationship between the newly characterized *Giardia* lineages and rodent hosts, suggesting rodents as important reservoirs of *Giardia* and its close relatives. These findings provide insights into the diversity of *Giardia* species and their public health potential in localities with human-wildlife interaction and further emphasizes the need for continued efforts to improve the molecular tools used to study microbial eukaryotes, especially those with zoonotic potential.

## Highlights

• We report on the occurrence and diversity of *Giardia* in wild rodents from New York.
• Molecular analysis of 55 *Giardia* samples revealed seven new lineages.
• The *Giardia* lineages found pose no public health threat and are likely host-adapted.
• We present a new PCR assay targeting the 18S gene for better species determination.

## Graphical abstract

**Figure:**
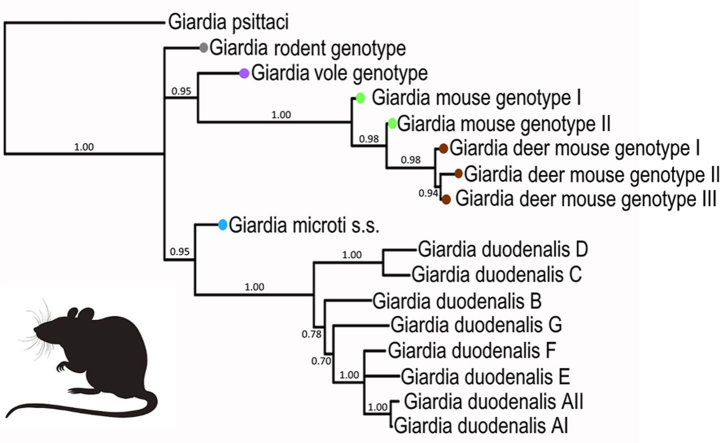

## 1 Introduction

*Giardia* is a genus of flagellated protozoans that parasitize the gastrointestinal tract of humans and wildlife worldwide. Currently, eight species of *Giardia* are considered valid based on morphology supplemented by molecular evidence: *G. muris, G. cricetidarum*, and *G. microti* in rodents, *G. ardeae* and *G. psittaci* in birds, *G. agilis* in amphibians, *G. peramelis* in quendas, and *G. duodenalis* (syn. *G. lamblia, G. intestinalis*), which has a broad host range that includes humans (Ryan et al., 2021). *G. duodenalis* is further sub-divided into eight genetic groups (called assemblages), which are commonly regarded as cryptic species within *G. duodenalis* and which has been supported by whole-genome sequencing of representative isolates as well as life history data such as host preference (Monis et al., 2009; Tsui et al., 2018; Seabolt et al., 2022; Wielinga et al., 2023). *G. duodenalis* assemblages A and B are the most well-studied lineages due to their high potential to cause large outbreaks of diarrheal illness in humans. *G. duodenalis* assemblages C through H and the remaining species in *Giardia* are understudied by comparison. In the last few years, the first reports of human cases caused by *Giardia duodenalis* sub-assemblage AIII, thought to be exclusively adapted to wild ruminants, as well as occasional zoonotic detections of other host-adapted assemblages of *G. duodenalis* (assemblages C, D, E, and F) have been documented (Pipiková et al., 2020; Garcia-R et al., 2021). While human cases of these assemblages have remained rare, these reports represent important findings suggesting the possibility of zoonotic potential of host-adapted *Giardia* species, especially in localities where humans and wildlife have increasingly common interactions. Detecting and characterizing relationships between host and parasite, including non-pathogenic or opportunistic taxa, are therefore important steps towards untangling transmission cycles and identifying potential risk factors and can provide additional evolutionary or epidemiological context to comparisons with pathogenic strains that are responsible for outbreaks.

Wild rodents are parasitized by four named species of *Giardia*: *G. microti* and *G. cricetidarum* in rodents belonging to the Cricetidae family, *G. muris* in primarily murine rodents (family Muridae), and the *G. duodenalis* assemblages A, B, and G (Helmy et al., 2018; Ryan and Zahedi 2019). Thus, rodents are parasitized by 50% of named *Giardia* species, and in addition, the genus *Octomitus* (itself the closest known sister to *Giardia*), is known primarily from rodents (Keeling and Brugerolle 2006; Seabolt et al., 2021). This would suggest that rodent hosts are important reservoirs of *Giardia* and its closest diplomonad relatives, both from an evolutionary perspective as well as epidemiologically. However, nearly all available data on the occurrence and distribution of *Giardia* originates from the *G. duodenalis* assemblages, while data from other species external to *G. duodenalis* remains very scarce despite the evidence that these organisms are common in areas of high human activity and indeed, outbreaks of giardiasis – e.g. “beaver fever”- have been linked to wild rodents (Prystajecky et al., 2015; Helmy et al., 2018; Tsui et al., 2018; Ryan and Zahedi 2019; Seabolt et al., 2021).

In this work, our objectives were to examine the occurrence, host specificity, and genotypic diversity of *Giardia* from wild rodents living within the New York City water supply watershed. We molecularly characterized 55 *Giardia* specimens using nested PCR assays targeting the *tpi, gdh, bg,* and *ef1α* loci, as well as introduced a newly developed PCR assay to amplify nearly complete sequences of the 18S rDNA gene for species-level classification. We present new molecular evidence suggesting that genotype diversity of rodent-derived *Giardia* spp. in the New York watershed is high, comprising at least seven novel lineages.

## 2 Materials and methods

### 2.1 Sampling and DNA extraction

DNA preparations obtained from wildlife scats determined to be positive for *Giardia* spp*.* were included in this study after being initially collected between 2006 through 2020 as part of a long-term watershed monitoring collaboration between the New York City Department of Environmental Protection (NYCDEP) and Centers for Disease Control and Prevention (CDC). Scat samples were collected by NYCDEP staff and identified to species level, with the exception of *Peromyscus* spp*.* (deer mice), which were identified to genus. All scat samples included in this study were from rodents, except one which was from a mallard duck and was collected alongside rodent scats. Additional details describing field collection and handling of wildlife scats are reported in Feng et al. (2007). All samples were shipped unpreserved on ice to the CDC for DNA extraction and PCR testing.

Total DNA was extracted using a Fast DNA spin kit for Soil (MP Biomedicals, Irvine, CA) according to manufacturer's instructions and kept at −80C for long-term storage. The presence of *Giardia* spp*.* was tested by qPCR amplification of a ∼65 bp portion of the 18S ssu-rDNA locus as described in Verweij et al. (2003) using 5 μL of template DNA. All samples were run in duplicate alongside molecular grade water as a negative control and a *G. duodenalis* assemblage B positive control identified from a human fecal specimen. A final set of 55 samples with averaged Ct values less than 30.0 were determined to be positive for *Giardia* spp. and selected for molecular characterization at the 18S ssu-rDNA, elongation factor 1α (*ef1a*), and subtyping loci *tpi, gdh,* and *bg*.

### 2.2 Molecular characterization of *Giardia* genotypes

PCR amplification of the 18S ssu-rDNA gene was achieved using a nested PCR assay designed by us to amplify nearly the entire gene (∼1500 bp in *Giardia*) in order to capture the maximum number of informative characters for species-level determination. A reverse primer, Gia18S-1468r (5′-CAGGTTCACCTACGGATACC-3′), was designed by manual alignment of complete 18S ssu-rDNA sequences of *G. muris*, *G. ardeae*, *G. peramelis*, and *G. duodenalis* and identifying a suitable binding site near the 3′ end of the locus. This primer was paired with the previously published forward primer RH11 (5′-CATCCGGTCGATCCTGCC-3’; Appelbee et al., 2003) for the primary PCR reaction, which was carried out using an Applied Biosystems (Foster City, CA) Proflex thermal cycler as follows: initial denaturation at 94ᵒC for 5 min; 35 amplification cycles of 94ᵒC for 45 s (s), annealing at 60ᵒC for 45s, extension at 72ᵒC for 90s, and lastly, a final extension step at 72ᵒC for 10 min. The primary PCR master mix consisted of 5 μL of 10X PCR Buffer (final concentration of 1X; GeneAmp PCR Buffer II, Applied Biosystems), 100 μM dNTPs, 2 μg/μL BSA, 2 mM MgCl2, 0.75 U Promega GoTaq DNA polymerase, 250 nM each of primers, either 2 or 5 μL template DNA based on sample type, plus molecular grade water for a final reaction volume of 50 μL. Because the 18S rDNA gene is sufficiently long that amplifying and sequencing it in one continuous fragment is challenging, we designed a nested primer, Gia18S-940f (5′-GTGGAGTCTGCGGCTCAAT-3′), based on a conserved region in the center of the 18S ssu-rDNA locus. This primer and its reverse complement (Gia18S-940r; 5′-ATTGAGCCGCAGACTCCAC-3′) were used along with the corresponding primary reaction primers (e.g. RH11f and Gia18S-940r; Gia18S-1468r and Gia18S-940f) in secondary PCR reactions to amplify approximately 1385 bp of the gene. The secondary nested PCR master mix consisted of 29.85 μL molecular grade water, 5 μL of 10X PCR Buffer, 100 μM dNTPs, 500 nM of secondary primers, 2 mM MgCl2, 0.75 U polymerase, 2 μL of primary PCR template and followed the same thermal cycling conditions as the primary reaction.

For *ef1α* nested PCR, the primary and secondary reactions were carried out using the same master mix volumes as 18S ssu-rDNA PCR, substituting primers GlongF (5′-GCTCSTTCAAGTACGCGTGG-3′) and EF1a-R (5′-AGCTCYTCGTGRTGCATYTC-3′) from Adams et al. (2004) for the primary reaction with cycling conditions of: initial denaturation at 94ᵒC for 5 min; 35 amplification cycles of 94ᵒC for 45s, annealing at 55ᵒC for 45s, extension at 72ᵒC for 60s, and final extension step at 72ᵒC for 7 min. Secondary reactions again followed master mix volumes for nested 18S ssu-rDNA reactions and primers GiaEf1a-121f (5′-GACCAGYTYAAGGACGAGCG-3′) and GiaEf1a-786r (5′-CTCBGAGGTCAAGTCYGTCGAG-3′), both designed in this study. Cycling conditions for the secondary reaction were identical to the primary *ef1α* reaction except for annealing at 59ᵒC.

PCR reactions for subtyping loci *tpi, gdh,* and *bg* followed previously published protocols in Cacciò et al. (2008) and Lalle et al. (2005), each using 2 μL of template. All samples tested by conventional PCR were run in duplicate, with *G. duodenalis* assemblage B DNA and molecular grade water acting as positive and negative controls respectively. Successful amplification was checked by gel electrophoresis on a 1.5% agarose gel. PCR-positive samples were sequenced in both directions using secondary PCR primers and BigDye v3.1 dideoxy chemistry on an Applied Biosystems 3500 genetic analyzer. Raw sequence reads were trimmed, assembled into contigs, and manually corrected using ChromasPro version 2.1.7, and when possible, overlapping 18S ssu-rDNA sequence fragments from the same sample were assembled into one contig.

### 2.3 Phylogenetic analysis

Assembled contigs were manually aligned against available representative sequences from *G. microti*, *G. ardeae*, *G. muris, G. peramelis, G. cricetidarum, G. psittaci*, and *G. duodenalis* assemblages A-G (incl. representatives from sub-assemblages AI and AII) per locus. A full list of Genbank accession numbers for the representative sequences used is given in Table S1. The 18S ssu-rDNA alignment contained only sequences for which both fragments of the gene were successfully sequenced and could be co-assembled into one contig. To capture species-level relationships, we generated a concatenated alignment of nearly complete 18S ssu-rDNA, *ef1a*, and *bg* sequences using representative *Giardia* spp*.* samples sequenced in this study, *G. microti* (from vole samples), and additional representatives of *G. duodenalis* assemblages A-G and *G. psittaci* as outgroups. We were not able to include additional loci in the concatenated alignment since we were unsuccessful in sequencing all loci from representative samples.

For all alignments, the best-fitting substitution model was estimated using jModelTest2 using the Akaike information criteria (AIC) (Darriba et al., 2012). Bayesian phylogenies were computed on the Aspen HPC cluster located at CDC, using MrBayes v3.2.2 with the selected evolutionary model and the best starting tree from jModelTest2 (Ronquist et al., 2012). Two simultaneous MrBayes runs consisting of 4 chains each with a heated temperature of 0.2 were run for 5 million generations to start, sampling trees every 1000 generations. The first 25% of trees were discarded as burn-in. The computation was stopped when the effective sample size was estimated to be greater than 200 and the standard deviation of split frequencies was less than 0.01, indicating convergence. Nodes were collapsed to polytomies when the posterior probability support was less than 0.70. The resulting phylogenies were visualized using the Interactive Tree of Life (iTOL) web portal (Letunic and Bork 2019; URL: https://itol.embl.de).

## 3 Results

### 3.1 PCR amplification success and intra-locus variation

#### 3.1.1 18S ssu-rDNA and ef1a loci

PCR amplification was overall low for all loci tested, with variable results per locus (summarized in Table 1 per host taxon). Our modified 18S ssu-rDNA assay relies on two separate secondary/nested PCRs to amplify approximately half of the locus each – 10 sequences of the 5′ fragment and 13 sequences of the 3’ fragment amplified successfully, with a total of 9/55 (16%) of samples tested able to be assembled into nearly complete contigs. Amplification success of *ef1a* was slightly more efficient, with 21/55 (38%) samples amplified. In both loci, we observed minor sequence heterogeneity among the individual sequences, primarily in hypervariable regions of the 18S ssu-rDNA locus and in synonymous (“silent”) sites in the *ef1a* reading frame. In these instances, IUPAC nucleotide ambiguity codes were used when we were unable to determine the correct nucleotide.

**Table 1:** PCR amplification success per host taxon.

Host		No. Samples Tested	No. samples sequenced per locus			
			18S^a^	*ef1α*	*tpi*	*bg*
Rodents		54	9	20	10	49
undet. rodent species		2	1	2	2	1
Muridae		8	2	3	0	4
	*Rattus norvegicus* (Norway rat)	4	0	0	0	3
	*Mus musculus* (house mouse)	4	2	3	0	1
Cricetidae		44	6	15	8	44
	*Microtus pennsylvanicus* (meadow vole)	4	1	4	3	4
	*Myodes gapperi* (boreal red-backed vole)	1	0	0	0	1
	*Peromyscus* spp. (deer mouse)	38	5	11	4	38
	*Ondatra zibethicus* (muskrat)	1	0	1	1	1

Birds		1	0	0	0	1
*Anas platyrhynchos* (mallard duck)		1	0	0	0	1

Total		55	9	21	10	50

a both fragments amplified from same sample.

#### 3.1.2 Subtyping loci tpi, gdh, and bg

We were unable to employ the common MLG subtyping method using partial sequences of the *tpi, gdh,* and *bg* genes here for *Giardia* spp. due to low amplification success of the *tpi* (10/55; 18%) and *gdh* (0 samples amplified) genes. An additional 8 samples showed inconclusive amplification at the *tpi* locus and could not be sequenced. Amplification of the *bg* locus was the most successful of any loci tested by us*,* with 50/55 (90%) of samples able to be sequenced (Table 1). Occasional instances of allelic sequence heterogeneity (double peaks) in the chromatograms of some *bg* sequences were observed. We again substituted IUPAC ambiguity codes when the correct nucleotide could not be determined.

### 3.2 Phylogenetic relationships among *Giardia* lineages

**Fig. 1:**
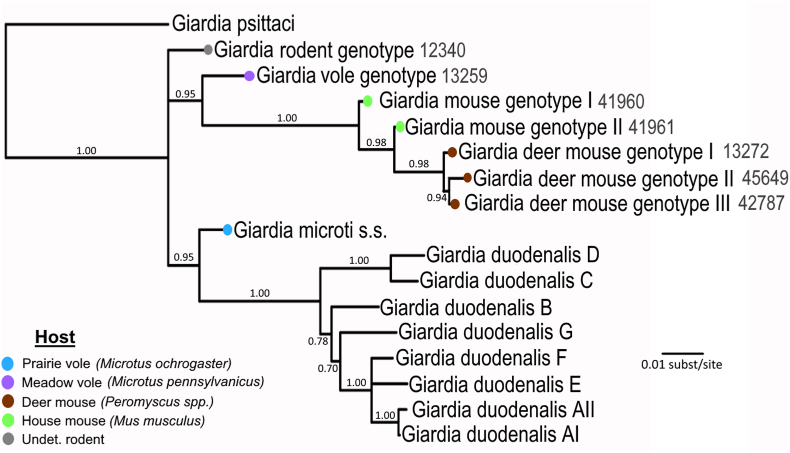
Species tree computed from a concatenated alignment of 18S ssu-rDNA, *ef1a*, and *bg* loci. Sample IDs for novel *Giardia* genotypes identified in this study are shown in grey text. Host information for the novel *Giardia* genotypes and *G. microti* are shown using colored nodes.

A multiple sequence alignment consisting of 2566 nucleotide residues was concatenated from representative 18S ssu-rDNA, *ef1a,* and *bg* sequences of *Giardia duodenalis* (assemblages A-G, *G. psittaci,* and individual *Giardia* spp*.* samples for which all three target genes had been successfully amplified and sequenced in this study, for a total of 17 composite sequences available for phylogenetic analysis. The resulting Bayesian phylogeny represented evolutionary relationships at the species level and placed *G. psittaci* external to all other sequences which arose from a single polytomic node with three strongly supported branches (Fig. 1). Of these, one branch contains a single, novel lineage of *Giardia* sp.; the second branch consists of six novel *Giardia* spp*.* lineages: one genotype from a meadow vole (*Microtus pennsylvanicus),* two genotypes from house mice (*Mus musculus*) and three genotypes from deer mice (*Peromyscus* spp.); and the third branch consisted of the *G. microti sensu stricto* lineage described in van Keulen et al. (1998) from voles and muskrats (GenBank acc. no. AF006676), which itself is sister to all *G. duodenalis* assemblages. Novel *Giardia* spp*.* were provisionally named following the host from which the sample originated and numbered sequentially in cases where multiple lineages were identified from the same host species.

The *G. duodenalis* assemblages are commonly acknowledged to each represent cryptic species based on whole-genome evidence, therefore we used this clade to estimate the suitability of the concatenated alignment of three gene loci to provide robust species-level discriminatory power. All *G. duodenalis* assemblages were recovered with strong statistical support and were separated by single nucleotide polymorphism (SNP) distances of 4–107 SNPs (99.84–94.37% nucleotide identity) and likewise, the SNP distance range between any of the 8 *G. microti* lineages represented was 8–111 SNPs (99.72–94.36% identity). Comparing all *G. duodenalis* assemblage sequences against *G. microti* and all novel *Giardia* spp. sequences*,* a range of 70 (*G. microti* s.s. and *G. duodenalis* asm. B; 97.12% identity) up to 175 SNPs (*G.* deer mouse genotype I and *G. duodenalis* asm. D; 91.78% identity) was recovered. SNP distances and percent identities are given in Table S2. When host information was overlaid onto the phylogeny, it was observed that the three species lineages from deer mouse hosts were most closely related, and similarly with the house mouse lineages, and lastly a third group consisting of the ‘rodent’ lineage (from an undetermined rodent host), the [meadow] vole lineage, and *G. microti* from a prairie vole. Thus, these composite lineages were provisionally named in sequential order by the host in which the genotype was first identified (e.g. deer mouse genotype I, deer mouse genotype II, etc.).

**Fig. 2:**
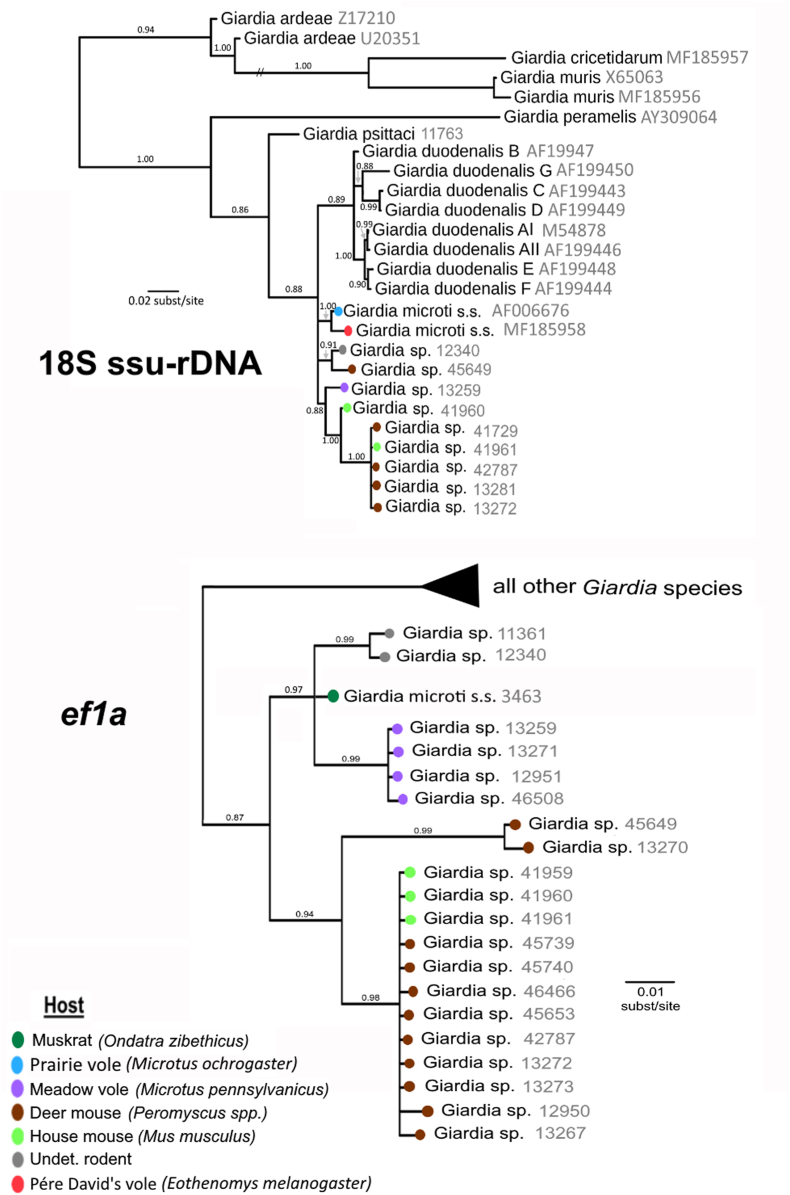
Gene trees of 18S ssu-rDNA (top) and *ef1a* loci (bottom). Host information is represented for both panels using colored nodes. Sample identifiers are shown in grey text.

Individual gene trees for the 18S ssu-rDNA and *ef1a* loci were analyzed in similar fashion. The 18S ssu-rDNA phylogeny again recovered a polytomy sister to *G. psittaci* that gave rise to multiple lineages of *Giardia* spp*., G. microti*, and the *G. duodenalis* clade (Fig. 2, top). All remaining *Giardia* lineages represented in the 18S ssu-rDNA analysis were recovered as ancestral to *G. psittaci* and the *G. microti/G. duodenalis/*novel *Giardia* spp*.* polytomy. The *ef1a* analysis recovered all novel lineages as a monophyletic sister clade to the remainder of *Giardia*, including *G. duodenalis*. This clade contains five strongly supported branches, which visually suggest potential patterns of host preference (Fig. 2, bottom). One group consists of two sequences that are both collected from undetermined rodent hosts, a second group contains four sequences from meadow voles, a third group contains two sequences from deer mouse hosts, a fourth group contains 12 sequences (9 from deer mouse hosts and 3 from house mouse hosts), and lastly, a single sequence from a muskrat.

#### 3.2.1 Intra-species relationships at bg and tpi subtyping loci

**Fig. 3:**
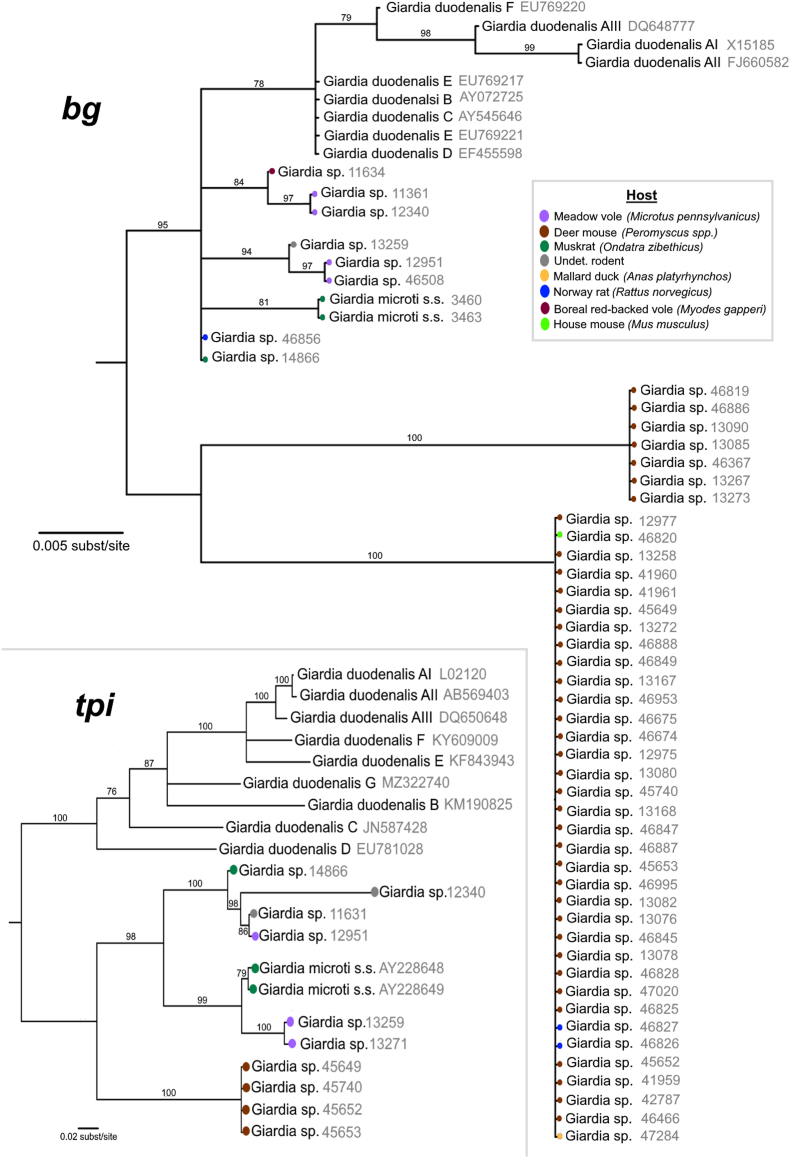
Gene trees of subtyping loci β-giardin (*bg*) and triose-phosphate isomerase (*tpi*; inset). Host information is represented for both panels using colored nodes. Sample identifiers are shown in grey text.

We identified 7 novel sequence types from the 475 bp alignment of *bg* sequences, five of which arise from a polytomy that also includes *Giardia microti* s.s. (from muskrats) and a clade containing all *G. duodenalis* assemblages/species complex (Fig. 3, top). Of these five lineages, four (incl. *G. microti*) are represented by a pair of sequences each, with the remaining two lineages represented by a single sequence each. An additional two well-represented lineages (n = 7 and 35 sequences) were also recovered external to the polytomic clade. In the absence of sufficiently characterized reference sequences, we were unable to reliably determine species identity for the novel subtypes. However, similar to the 18S ssu-rDNA and *ef1a* loci, visual observation of host origin per sequence overlaid on the phylogeny again suggests moderate to strong host-specific associations for most subtypes, with the two clades external to the polytomy primarily sampled from deer mice (*Peromyscus* spp.) hosts, but also containing sequences sampled from house mouse, Norway rat, and a mallard duck (the only non-rodent host). The polytomic lineages were represented mostly by vole and muskrat hosts, and 1 Norway rat, deer mouse, and an undetermined rodent host.

Five novel sequence types were recovered from analysis of the *tpi* alignment containing 496bp, all of which formed a monophyletic clade that also contained *G. microti* s.s., and which was external to the *G. duodenalis* clade (Fig. 3, bottom). Within this clade, we identified two lineages: one containing only sequences sampled from deer mouse hosts, and the other splitting into smaller clades each containing sequences collected from muskrat, vole, or undetermined rodent hosts.

### 3.3 Distribution of host species by *Giardia* sequence types

Among the composite genotypes, as well as loci examined individually, the most ancestral split between the *G. duodenalis* clade, *G. microti,* and the novel lineages is characterized by host species belonging to the Cricetidae family of rodents (New World rats and mice, voles, lemmings, hamsters, and muskrats). *Giardia* lineages derived from Old World rats and mice (family Muridae) consistently arise from within this broader cricetid clade (Fig. 1, Fig. 2, Fig. 3). When examined per locus, a majority of subtypes were identified from only one host species (n = 16/23 types, Table 2). Among subtypes with multiple hosts identified, 4 were found in hosts belonging to both the Cricetidae and Muridae families, indicating less strict host preference or ongoing genetic recombination between lineages at these loci (or both).

**Table 2:** Distribution of host taxa per *Giardia* subtypes.

Locus	Subtype	Hosts identified in this study (no. sequences analyzed)
18S^a^	18S-1	undet. rodent (1), deer mouse (1)
	18S-2	meadow vole (1)
	18S-3	house mouse (1)
	18S-4	house mouse (1), deer mouse (4)
	*G. microti s.s.*	prairie vole (1), Pére David's vole (1)
*ef1α*	*ef1α-1*	undet. rodent (2)
	*ef1α-2*	meadow vole (4)
	*ef1α-3*	deer mouse (2)
	*ef1α-4*	house mouse (3), deer mouse (9)
*tpi*	*tpi-1*	muskrat (1)
	*tpi-2*	undet. rodent (1)
	*tpi-3*	undet. rodent (1), meadow vole (1)
	*tpi-4*	meadow vole (2)
	*tpi-5*	deer mouse (4)
	*G. microti s.s.*	muskrat (2)
*bg*	*bg-1*	boreal red-backed vole (1)
	*bg-2*	meadow vole (2)
	*bg-3*	undet. rodent (1)
	*bg-4*	meadow vole (2)
	*bg-5*	Norway rat (1), muskrat (1)
	*bg-6*	deer mouse (7)
	*bg-7*	house mouse (1), Norway rat (2), mallard duck (1), deer mouse (30)
	*G. microti s.s.*	muskrat (2)

a both fragments amplified from same sample.

## 4 Discussion

Expanding the breadth of molecular characterization tools available for microbial eukaryotes, particularly those with known or suspected zoonotic potential, is of crucial importance to public health activities and environmental monitoring. Many microbial eukaryotes, *Giardia* a salient example among them, contain both human pathogenic and non-pathogenic lineages that are morphologically indistinguishable, requiring the use of high-resolution molecular methods to differentiate between species and strains (=subtypes). Comparing the biology and relationships of opportunistic or commensal (i.e. non-pathogenic) strains to pathogenic strains can provide valuable insights into key biological differences of interest to public health scientists, such as differential virulence, zoonotic potential, transmission dynamics, or other epidemiological risk factors.

Rodents play host to a diverse number of *Giardia* species (*G. duodenalis* assemblages AI, *G. hominis*/sub-assemblage AII, B, and G; *G. muris*, *G. microti*, and *G. cricetidarum*. In addition, *Octomitus* spp. are closely related to *Giardia* and likewise are only known to parasitize rodent species. All novel *Giardia* spp. genotypes reported in this study were identified from rodent hosts belonging to the Muridae or Cricetidae families, with a single *bg* sequence obtained from a mallard duck. These new data contribute to growing evidence suggesting that rodents are among the most important natural reservoirs of parasitic diplomonads (van Keulen et al., 2002; Helmy et al., 2018; Ryan et al., 2021; Seabolt et al., 2021). No reports of human cases outside of the *Giardia duodenalis* assemblages have been identified, however the high diversity of *Giardia* spp. in rodent hosts is important due to rodents’ frequent interactions with humans as common household pests in both urban and rural settings. Our results suggest that these new *Giardia* genotypes may be of little, if any, concern to the water quality within the watersheds from which they were sampled, however the robustness of this conclusion should be verified by case data and additional testing of water specimens.

Our phylogenetic analysis revealed seven lineages of *Giardia*, six of which are previously uncharacterized and one which tentatively matched a European genotype first reported in Helmy et al. (2018) at the 18S locus, establishing that two allopatric sets of genotypes exist among wild rodents in North America and Europe. Our conclusion is that the novel genotypes we report here are likely distinct species of *Giardia*, however we have refrained from formally elevating them to species rank in this work since whole-genome sequences are not available to fully resolve the phylogenetic relations among these lineages and because the degree of host specificity remains to be characterized more thoroughly as supporting criteria for species rank.

### 4.1 Utility of new 18S ssu-rDNA nested PCR and genotyping techniques in Giardia

Molecular tools capable of both detecting and distinguishing *Giardia* sequences have variable success both in terms of PCR amplification success at the laboratory bench and with respect to their differentiating power. At the species level, several assays targeting fragments of the 18S ribosomal DNA (18S ssu-rDNA) have been developed, however the most widely used PCR assay targeting this locus in *Giardia* only amplifies a 285 bp region, which is insufficient for discriminating between closely related lineages such as *G. duodenalis* assemblages A and F, or the sub-assemblages of assemblage A, which have demonstrable differences in host-preference (Seabolt et al., 2022; Wielinga et al., 2023). Within species and assemblages, a multilocus typing approach using fragments of three housekeeping genes - triose-phosphate isomerase (*tpi*), glutamate dehydrogenase (*gdh*), and β*-*giardin (*bg*) – is widely employed, albeit with variable resolving power depending on the *G. duodenalis* assemblage identified (Caccio and Ryan, 2008; Sprong et al., 2009).

This study describes a new nested PCR assay which captures nearly all of the *Giardia* 18S ssu-rDNA locus by amplifying and sequencing two overlapping fragments in the secondary PCR. In this manner, our assay provides suitable differentiating power for species-level genotyping versus older assays which have lower phylogenetic resolution among very closely related lineages such as the sub-assemblages of assemblage A, and which are debated to be cryptic species themselves (Seabolt et al., 2022; Wielinga et al., 2023). The PCR primers were designed with specificity to *Giardia* and sequences of other diplomonad relatives to avoid non-specific amplification. Preliminary trials with *Octomitus* samples negative for *Giardia* confirmed no cross-reactivity. As has been reported previously for many PCR assays targeting *Giardia*, the amplification efficiency we report in this study is overall low, which may be the result of DNA degradation from 10 to 15-year-old samples (Thompson and Ash, 2016). Amplification success may improve with further optimizations to PCR conditions to better amplify long fragments. We also note that we did not detect any *G. muris* genotypes in any of our samples, which may be due in part to diminished sensitivity of the Verweij qPCR assay used for initial screening, as the reverse primer and probe have mismatches against the corresponding binding site in the *G. muris* 18S locus.

Among the subtyping loci, the *bg* nested PCR appears to work well for detecting and sequencing *Giardia* spp. from rodents. *Tpi* was also successful (but with low efficiency), however we were unable to amplify *gdh* fragments for any sample using previously published primers and assays. Similar results were reported for the *gdh* locus in Helmy et al. (2018).

### 4.2 Implications for taxonomic proposals in Giardia

New taxonomic proposals or revisions in *Giardia* must be able to be systematically applied to closely related species or clades, including ones that will be discovered and catalogued in the future through additional wildlife sampling. Our results show that using 18S ssu-rDNA or *ef1α* sequences alone, two conserved eukaryotic genes often used as species-level identifiers or “barcode” genes, did not provide adequate phylogenetic signal to fully resolve evolutionary relationships due to high sequence conservation. Likewise, housekeeping genes like *tpi, gdh,* and *bg* each have differing and sometimes discordant phylogenetic signals, perhaps as a result of recombination between co-occurring genotypes, making them unsuitable for species delineation in the absence of additional genes. Whole-genome sequencing, therefore, should be the objective for studies describing new diversity whenever possible.

No genome sequence has yet been published for *Giardia microti*, and when one is eventually sequenced, care should be taken to ensure it belongs to the correct taxon, as we have revealed here a number of lineages similar to *G. microti*. We strongly suggest that new species descriptions of *Giardia* should be accompanied by a genome sequence from holotype material so that taxonomic debates can easily be settled, unlike the debate that has centered on the *G. duodenalis* assemblages (Monis et al., 2009; Plutzer et al., 2010; Thompson and Ash, 2016; Seabolt et al., 2022; Wielinga et al., 2023). Here, our attempts to isolate material for WGS using anti-*Giardia* Dynabeads (IDEXX) were unsuccessful, however, the inability to isolate cysts may also be due to sample age and quality, incompatible antibodies, or preservation methods. Additionally, cesium chloride gradient flotations using specific gravities of 1.15 g/mL did not yield suitable material for sequencing, again attributable to the age of samples and/or preservation techniques. Unfortunately, the samples included in this report contained very little original material (less than a gram in some cases) and thus attempts to amplify enough DNA for WGS are limited. Modifications to the abovementioned techniques or metagenomic approaches may yield a viable method for direct sequencing of *Giardia* isolates such as these and should be a focus of future work.

## 5 Conclusions

This study characterized new diversity in *Giardia,* sampled from rodent hosts as part of a long-term New York watershed monitoring project. This report expands the number of known *Giardia* genotypes, effectively doubling the breadth of *Giardia* diversity found in rodents. The data do not suggest any threat to public health and are hypothesized to be host-adapted, commensal lineages. Continued efforts toward characterizing non-pathogenic or environmental strains such as we report here, using conventional or genomic methods, can provide additional evolutionary or epidemiological context to comparisons with pathogenic strains that are responsible for outbreaks, especially those with suspected zoonotic origins, and should be the subject of future efforts.

## Disclaimers

The findings and conclusions in this report are those of the authors and do not necessarily represent the views of the Centers for Disease Control and Prevention. This research did not receive any specific grant from funding agencies in the public, commercial, or not-for-profit sectors.

## Data availability

Sequences generated as part of this study have been submitted to GenBank and are available as accession numbers PQ318213 - PQ318237 (*tpi*), PQ318238 - PQ318259 (*ef1α*), PQ318260 - PQ318314 (*bg*), and PQ317783 – PQ317791 (18S ssu-rDNA).

## CRediT authorship contribution statement

**Matthew H. Seabolt:** Writing – review & editing, Writing – original draft, Visualization, Supervision, Methodology, Formal analysis, Data curation, Conceptualization. **Kerri A. Alderisio:** Writing – review & editing, Resources, Project administration. **Lihua Xiao:** Writing – review & editing, Funding acquisition. **Dawn M. Roellig:** Writing – review & editing, Supervision, Project administration.

## Declaration of competing interest

The authors declare no conflicts of interest.
